# Effect of introducing training in assessment tools for foundation trainees (F2) in intensive care and anaesthesia in a UK teaching hospital

**DOI:** 10.1186/cc9889

**Published:** 2011-03-11

**Authors:** A Raithatha, A Khaliq, P Prashast, D Bryden

**Affiliations:** 1 Northern General Hospital, Sheffield, UK

## Introduction

A 2-year F2 programme was implemented nationally in the UK in 2005. The curriculum consists of core competencies against which trainees are assessed, with a syllabus setting out specific knowledge, skills and attitudes to develop. An essential component of this curriculum is that trainees must meet specific objectives in relation to recognition and treatment of the acutely ill. Assessment tools used are: MSF (multisource feedback), Mini-CEX (clinical evaluation), DOPS (direct observation of procedural skills), and CbD (case-based discussion). Specific training programmes were introduced in 2008 to assist staff with conducting these assessments, as trainees had reported difficulty in completing them. Training was delivered using mixed methods of face-to-face contact backing up e-learning. Aims were to assess the number and grade of medical staff involved in assessment; to assess their willingness to be involved in F2 training and any barriers existing; to assess the degree of training and understanding of assessment tools; and to compare with historical data.

## Methods

The Modernising Medical Careers website [1] was used to create a questionnaire. Data were analysed retrospectively and results compared with those from a previous survey, conducted within our department in 2006.

## Results

Comparisons (bracketed) are with 2006 data. Sixty-four completed forms were returned, representing 51% of those surveyed. A total of 87.5% (80%) were involved in teaching and 68% (42%) in assessment of F2 s, with 66% (61%) being consultants. Seventy-six per cent felt that those involved in assessment should have specific training with 72% having received such training, compared with 42% in 2006. Twenty-two per cent would not assess an F2 if approached, with the majority (57%) citing lack of specific training as the reason. Twenty-seven per cent (48%) of those involved in assessment had not received any specific training. Of those who had been trained, all respondents had at least some knowledge of DOPS, Mini-CEX and CbD.

## Conclusions

Introduction of training has improved participation in both assessment and teaching, in addition to highlighting the need for those who were untrained not to undertake assessments they had not been trained to do. There is now a good understanding of assessment tools although further training is warranted to emphasise the valuable role of critical care experts in delivering training and assessment to foundation doctors.
